# Differential expression of follistatin and FLRG in human breast proliferative disorders

**DOI:** 10.1186/1471-2407-9-320

**Published:** 2009-09-09

**Authors:** Enrrico Bloise, Henrique L Couto, Lauretta Massai, Pasquapina Ciarmela, Marzia Mencarelli, Lavinia E Borges, Michela Muscettola, Giovanni Grasso, Vania F Amaral, Geovanni D Cassali, Felice Petraglia, Fernando M Reis

**Affiliations:** 1 Departments of Obstetrics & Gynecology and Physiology, Federal University of Minas Gerais, Belo Horizonte, Brazil; 2 Department of Obstetrics and Gynecology, University of Siena, Siena, Italy; 3 Department of Biomedical Sciences, University of Siena, Siena, Italy; 4 Department of Molecular Pathology and Innovative Therapies, Polytechnic University of Marche, Ancona, Italy; 5 Department of Physiology, University of Siena, Siena, Italy; 6 Department of Pathology, Federal University of Minas Gerais, Belo Horizonte, Brazil

## Abstract

**Background:**

Activins are growth factors acting on cell growth and differentiation. Activins are expressed in high grade breast tumors and they display an antiproliferative effect inducing G0/G1 cell cycle arrest in breast cancer cell lines. Follistatin and follistatin- related gene (FLRG) bind and neutralize activins. In order to establish if these activin binding proteins are involved in breast tumor progression, the present study evaluated follistatin and FLRG pattern of mRNA and protein expression in normal human breast tissue and in different breast proliferative diseases.

**Methods:**

Paraffin embedded specimens of normal breast (NB - n = 8); florid hyperplasia without atypia (FH - n = 17); fibroadenoma (FIB - n = 17); ductal carcinoma *in situ *(DCIS - n = 10) and infiltrating ductal carcinoma (IDC - n = 15) were processed for follistatin and FLRG immunohistochemistry and *in situ *hybridization. The area and intensity of chromogen epithelial and stromal staining were analyzed semi-quantitatively.

**Results:**

Follistatin and FLRG were expressed both in normal tissue and in all the breast diseases investigated. Follistatin staining was detected in the epithelial cytoplasm and nucleus in normal, benign and malignant breast tissue, with a stronger staining intensity in the peri-alveolar stromal cells of FIB at both mRNA and protein levels. Conversely, FLRG area and intensity of mRNA and protein staining were higher both in the cytoplasm and in the nucleus of IDC epithelial cells when compared to NB, while no significant changes in the stromal intensity were observed in all the proliferative diseases analyzed.

**Conclusion:**

The present findings suggest a role for follistatin in breast benign disease, particularly in FIB, where its expression was increased in stromal cells. The up regulation of FLRG in IDC suggests a role for this protein in the progression of breast malignancy. As activin displays an anti-proliferative effect in human mammary cells, the present findings indicate that an increased FST and FLRG expression in breast proliferative diseases might counteract the anti-proliferative effects of activin in human breast cancer.

## Background

Activins (A, B and AB, composed respectively of two βA subunits, two βB subunits and one βA and one βB subunit) are pleiotropic secreted polypeptides. They belong to the transforming growth factor-β (TGF-β) superfamily and exhibit regulatory roles over important cell cycle events such as cell proliferation, differentiation, apoptosis and consequently tumor growth [1]. Previously, we reported activin/inhibin localization in human breast [2] and increased expression of dimeric activin A in breast cancer tissue homogenates [3]. Importantly, we also demonstrated that in postmenopausal women with breast cancer, activin A levels in serum were increased and subsequently decreased after mastectomy, suggesting that a major source had been removed [3]. Despite this, activin's signal transduction components are down-regulated in high-grade breast cancer at protein level, as well as activin βB subunit, demonstrating that even though activin A is abundantly available in breast carcinoma, its transduction pathway is decreased [4]. Local inhibins are not increased either [2,4], leading to the question of why activin A is unable to induce its signaling pathway and, consequently, perform its biological functions. Additionally, activins display an antiproliferative effect in human breast cancer cells by arresting them in the G0/G1 cell cycle phase [5,6], an effect that may be counteracted by estrogen, since a crosstalk between activin and estrogen has been demonstrated [7].

Activins signal through cell-surface receptors that display predicted serine/threonine kinase activity. Activins bind selectively to ActRIB and ActRIIA receptors, while ActRIA and ActRIIB have been demonstrated to bind promiscuously to other TGF-β components [8,9]. The receptor-activated Smads 2 and 3 bind to the common smad 4, forming a complex that moves towards the nucleus, where it can modulate gene expression and promote activins biological effects [10]. These biological events are inhibited mainly by the glycosylated single-chain proteins, follistatin (FST) and follistatin-related protein, encoded by follistatin-related gene (FLRG) [10]. Besides activins, FST also binds and neutralizes other TGF-β growth factors such as myostatin and bone morphogenetic proteins (BMPs) 2, 4, 6 and 7 [11-13], while FLRG has been demonstrated to neutralize myostatin [14,15].

Recently, a number of reports showed that the activin/FST system plays important roles in the progression of malignant diseases by regulating cell proliferation or angiogenesis [16]. Coherently with activin's role in breast carcinogenesis, FST and FLRG have also been associated with tumorigenesis of different tissues [17-19]. Specifically in the mammary gland of immunodeficient SCID mice, FST-expressing R30C tumor displayed increased angiogenesis but a higher susceptibility to undergo serum starvation-induced apoptosis and therefore limiting tumor progression properties [20]. In high grade infiltrating ductal carcinomas (IDC), FST expression pattern was not altered, while FLRG expression was increased in the same tumor. Moreover, FLRG silencing through small interfering RNA (siRNA) induced significant tumor growth inhibition, an effect that was reversed upon the addition of exogenous FLRG [17], demonstrating that FLRG antagonizes activin effects in neoplastic cells.

In order to determine whether there is an aberrant expression of FLRG at different stages of tumor progression and also to determine if FST is involved in human breast tumor progression, we investigated the expression profile of activin binding proteins in different human breast proliferative diseases. Specifically, the aim of the present study was to define the expressional pattern of FST and FLRG in normal breast (NB) and in different cases of breast proliferative diseases such as: florid hyperplasia without atypia (FH), fibroadenoma (FIB), ductal carcinoma *in situ *(DCIS) and infiltrating ductal carcinoma (IDC).

## Methods

### Subjects

The breast samples used in this study were collected from 67 patients with ages ranging from 29 to 72 years (Table 1) regardless of the menstrual cycle phase or menopausal status, diagnosed between 2004 and 2006 in the Hermes Pardini Laboratories Pathology Service (Belo Horizonte, MG, Brazil). We included cases of normal breast (NB, n = 8), florid hyperplasia without atypia (FH, n = 17), fibroadenoma (FIB, n = 17), ductal carcinoma *in situ*, grades 1, 2 and 3 (DCIS, n = 10) and infiltrating ductal carcinoma, grades 1, 2 and 3 (IDC, n = 15). Breast lesions were diagnosed by the pathology service of the same institution and the study was carried out with the permission of the Ethics Committee of the Federal University of Minas Gerais.

**Table 1:** Histological characterization of the breast proliferative diseases

Cases	Age range	Number of cases
*Normal breast *(**NB**)	29 - 67	8
*Florid hyperplasia without atipia *(**FH**)	32 - 71	17
*Fibroadenoma *(**FIB**)	30 - 60	17
*Ductal carcinoma *in situ (**DCIS**)	29 - 69	10
*Infiltrative ductal carcinoma *(**IDC**)	41 - 72	15

### Immunohistochemistry

Immunohistochemistry (IHC) was performed on 5-μm sections that were mounted on gelatinized slides, deparaffinized and rehydrated through graded concentrations of ethanol, followed by endogenous peroxidase blockage by the use of 3% H_2_O_2 _in methanol solution. Then, sections were washed in PBS and incubated with normal rabbit serum for 1 h to block non-specific binding sites. The sections were incubated with the primary antibodies in 0.1% BSA/PBS at 4°C overnight at a final dilution of 1:200. Primary polyclonal antibodies raised against human antigens used in the present experiment were: rabbit anti-FST kindly donated by Dr. Wylie Vale (Salk Institute, La Jolla, USA) as previously described [21-23] and rabbit anti-FLRG kindly donated by Dr Véronique Maguer-Satta (Centre Léon Bérard, Lyon, France) [21,22,24,25]. Subsequently, biotinylated secondary antibodies were added to the sections during 30 min followed by peroxidase streptavidin incubation in ABC reagent (Vectastain Elite Universal Kit - Vector Laboratories, Burlingame CA, EUA). Diaminobenzidine (DAB - Sigma Chemicals. CO, St Louis, MO, USA) was successively used in order to visualize the immunolocalization of the primary antibodies. Tissue slices were counterstained with hematoxylin (Sigma Chemicals). Negative control slices were incubated with normal serum instead of primary antibodies.

### Probe preparation

FST and FLRG in situ hybridization probes [21,22,25] were prepared according to the procedure previously described [26] and modified [27]. cDNA obtained from healthy endometrium was amplified by PCR using 200 ng of primers for FST, FLRG, and the internal control genes β-actin and GAPDH, all presented in Table 2. FST, FLRG, β-actin and GAPDH amplicons were then labelled with the digoxigenin (Dig), Dig-labelled dUTP (Dig-11-UTP, Boehringer Mannheim, Mannheim, Germany).

**Table 2:** Primers and probes used for the in situ hybridization

Gene	Primer sequence	Product size
*FST*	5'-TGCCACCTGAGAAAGGCTAC-3' 5'-ACAGACAGGCTCATCCGACT-3'	201 bp
*FLRG*	5'-ACCTGAGCGTCATGTACCG-3' 5'-TGTGGCACGAGGAGATGTAG-3'	198 bp
*B-actin*	5'-GAT CAT TGC CCT CCT GAG C-3 5'-CAC CTT CAC CGT TCC AGT TT-3'	308 bp
*GAPDH*	5'-ATG GGG AAG GTG AAG GTC GG-3' 5'-TGG TGA AGA CGC CAG TGG AC-3'	300 bp

### In situ hybridization

In situ hybridization (ISH) was performed according to modifications of the method previously described [26]. Briefly, the sections were dewaxed, rehydrated, fixed in 4% paraformaldehyde (for 15 min) and predigested with proteinase K (3 mg/ml in Tris-EDTA - Boehringer Mannheim) (for 12 min at 37°C). After acetylation in 0.25% acetic anhydride in triethanolamine, permeabilization with (0,1 M, pH 7.4) phosphate buffer saline (PBS)/Triton (X100) and ethanol dehydration, slides were ready to be hybridized. The sections were incubated with hybridization solution, which contained approximately 13 ng/ml DIG-labelled probes in hybridization buffer (30% of 100% deionized formamide - Boehringer Mannheim), 20% 20×SSC, 20% TE, 10% enatured salmon sperm DNA (10 mg/ml, Boehringer Mannheim), 1% tRNA (100 mg/ml; Boehringer Mannheim), and 14% H_2_O. The hybridization solution was incubated at 100°C for 5 min, cooled on ice for 10 min and incubated at 42°C overnight. Subsequently, for the immunoperoxidase staining, the sections were incubated with mouse anti-digoxigenin antibodies (2 μg/ml; Boehringer Mannheim) in PBS-3% BSA for 1 h. After washing in PBS/0,1% Tween 20, the sections were incubated in rabbit anti-mouse antibodies coupled to peroxidase (13 μg/ml; DAKO, Milan, Italy) in PBS with 3% BSA for 30 min. To enhance staining, sections were incubated with swine antibodies against rabbit immunoglobulins coupled to peroxidase (26 μg/ml; DAKO). The immunoreactivity was visualized by DAB chromogen. Negative control procedures were performed to assess the specificity of the ISH signal: 1) omitting Dig-labeled probe; 2) omitting anti-Dig antibody; 3) omitting Dig-labeled probe and anti-Dig antibody. Slides were counterstained with hematoxylin, dehydrated with alcohol, cleared with xylene and mounted with a coverslip before observation.

### Semiquantitative scoring

Images were acquired and analyzed on a Carl Zeiss Axioplan 2 imaging microscope by AxioCam HR CCD camera and AxioVision 3.1 software (Carl Zeiss, Göttingen, Germany) by three different researchers blinded to patients. For both in situ hybridization and immunohistochemistry, the intensity of epithelium chromogen staining was graded semiquantitatively on a scale of 0-3 arbitrary units with 0 indicating no detectable staining, 1 = weak, 2 = moderate and 3 = strong. The area with positive staining was analyzed using the following scale: 0 = no detectable staining; 1 = up to 10%; 2 = 10-50% and 3 = more than 50% of staining.

### Statistical analysis

The area and intensity of IHC and ISH staining for FST and FLRG are presented as medians and ranges, and differences between groups were assessed by Kruskal-Wallis analysis of variance followed by Dunn's test for non-parametric multiple comparisons.

## Results

**Figure 1:**
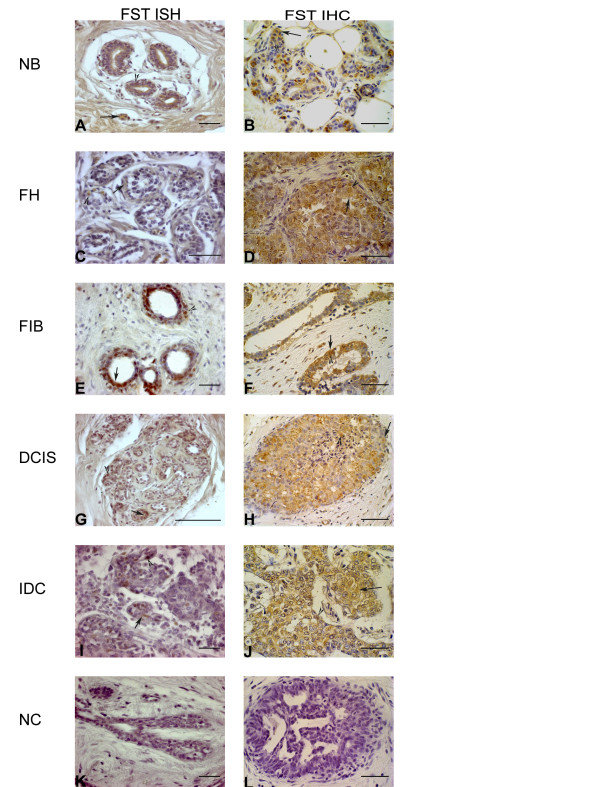
**Follistatin representative microscopic views of: mRNA expression (A, C, E, G, I) as detected by in situ hybridization (ISH); and protein expression (B, D, F, H, J) as identified by immunohistochemistry (IHC) for normal breast (NB), florid hyperplasia (FH), fibroadenoma (FIB), ductal carcinoma *in situ *(DCIS) and invasive ductal carcinoma (IDC)**. FST staining was detected in the epithelial cytoplasm (arrow) and nucleus (arrowhead) in the normal, benign and malignant breast tissue, with a stronger staining in the FIB peri-alveolar stromal cells. Negative controls were performed omitting anti-Dig antibody; Dig-labelled probe and anti-Dig antibody for ISH (**K**) and omitting the primary antibody for IHC (**L**). *Bar *50 μm.

**Figure 2:**
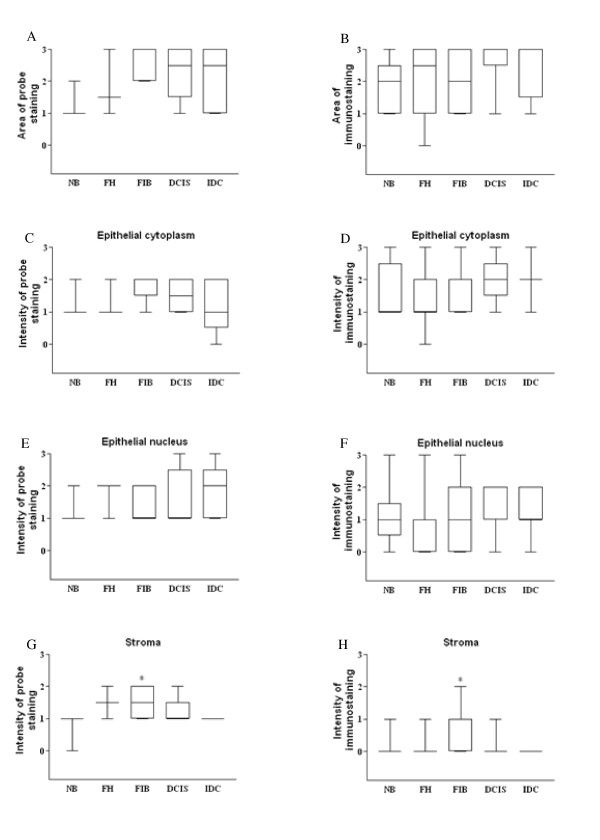
**FST diagrammatic presentation of semi-quantitative data for normal, benign and malignant breast tissue**. FST expression was analyzed by in situ hybridization (**A**,**C**,**E**,**G**) and immunohistochemistry (**B**,**D**,**F**,**H**,) in the normal breast (**NB**), florid hyperplasia (**FH**), fibroadenoma (**FIB**), ductal carcinoma *in situ *(**DCIS**) and infiltrative ductal carcinoma (**IDC**). FST stromal intensity was stronger in the FIB; *p < 0.05 compared to NB (Kruskal-Wallis followed by Dunn's test).

**Figure 3:**
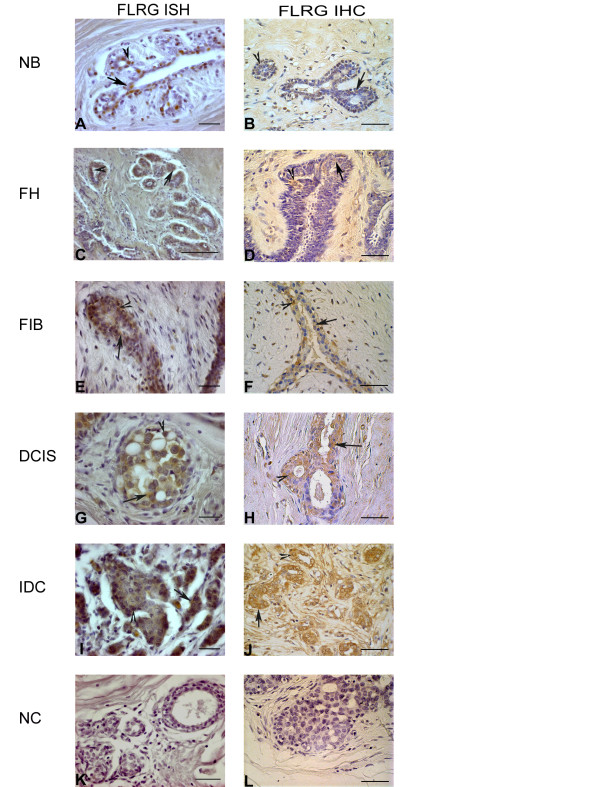
**FLRG representative microscopic views of: **mRNA (**A**,**C**,**E**,**G**,**I**) and protein (**B**,**D**,**F**,**H**,**J**) expression detected by in situ hybridization (**ISH**); and immunohistochemistry (**IHC**), respectively, in normal breast (**NB**), florid hyperplasia (**FH**), fibroadenoma (**FIB**), ductal carcinoma *in situ *(**DCIS**) and infiltrative ductal carcinoma (**IDC**). FLRG staining was detectable in the epithelial cytoplasm (arrow) and nucleus (arrowhead) in all the cases analyzed, with some weak staining detected in the stromal cells. **K **and **L: **negative controls for ISH and IHC respectively. *Bar *50 μm.

**Figure 4:**
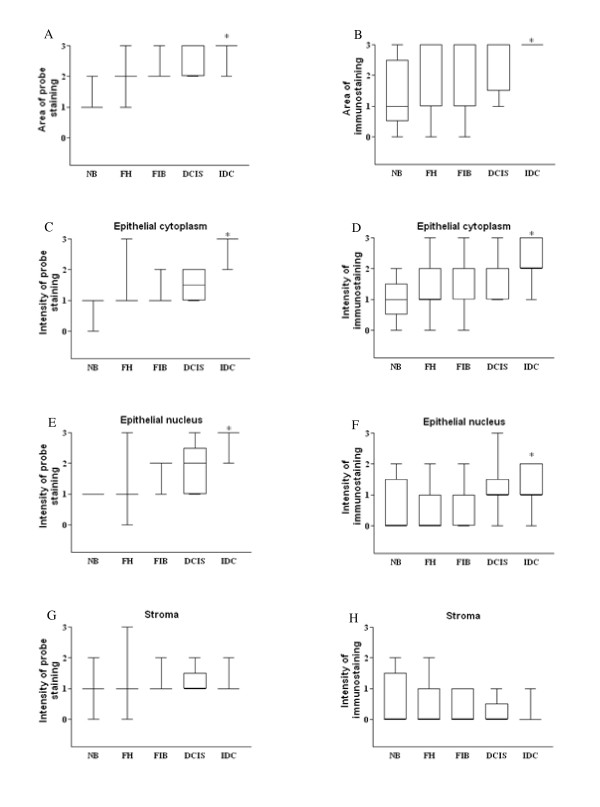
**FLRG diagrammatic presentation of semi-quantitative data for normal, benign and malignant breast tissue**. FLRG expression was analyzed by in situ hybridization (**A**,**C**,**E**,**G**) and immunohistochemistry (**B**,**D**,**F**,**H**,) in normal breast (**NB**), florid hyperplasia (**FH**), fibroadenoma (**FIB**), ductal carcinoma *in situ *(**DCIS**) and infiltrative ductal carcinoma (**IDC**). FLRG area of staining was augmented in the IDC, correspondingly, epithelial cytoplasm and nuclear staining was stronger in the IDC; *p < 0.05 compared to NB (Kruskal-Wallis followed by Dunn's test).

Clinical samples of the breast proliferative diseases were classified by the consultant pathologist as indicated in the Table 1. In order to determine cellular localization and the expressional profile of FST and FLRG in the normal breast and in different breast proliferative diseases, the tissue structures that were analyzed for mRNA and protein staining were: epithelial cytoplasm, epithelial nuclei and stromal tissue. Tissue localization and staining patterns of FST mRNA and protein are illustrated in Figure 1, with the corresponding semiquantitative analyses depicted in Figure 2. The same is shown for FLRG Figure 3 and Figure 4. FST and FLRG staining localization and patterning were similar for mRNA and protein, demonstrating that transcription and translation were in accordance, and no differences between the three diverse grades of DCIS and IDC analyzed were identified (data not shown).

### FST expression and tissue distribution

Less than 10% of the cells were stained by the FST probe in the NB group, while for the breast proliferative disorders, 10-50% of the cells were positive for the probe staining. FST protein staining was scored between 10-50% for all the groups analyzed and no differences in the area of staining were observed for FST in the different stages of tumor progression examined (Figure 2A and 2B). In all groups both epithelial cytoplasm and epithelial nucleus staining were homogeneously distributed throughout the epithelial cells, with a moderate to weak staining pattern, although epithelial nucleus staining was undetected in some cases at the protein level (Figure 2F). No differences of FST intensity staining were detected for the epithelial cytoplasm and epithelial nucleus (Figure 2C and 2F). The stromal staining was weak or undetectable for all the groups but FIB (Fig 1E and 1F), which showed an increased intensity of staining at both mRNA (P = 0.033) (Figure 2G) and protein (P = 0.032) (Figure 2H) level, when compared to the NB group. The negative controls of breast tissue showed no staining (Figure 1K and 1L).

### FLRG expression and tissue distribution

FLRG staining was present in NB breast tissue and in all breast proliferative diseases analyzed. FLRG staining at mRNA (Figure 4A) and protein (Figure 4B) level was present in less than 10% of the cells in the NB group and in 10-50% of the cells in the cases of FH, FIB and DCIS. In IDC cases, probe and immunostaining was present in more than 50% of the cells, thus demonstrating a significant increase when compared to the NB group (P = 0.015 and P = 0.017, for mRNA an protein staining, respectively). Both epithelial cytoplasm and epithelial nucleus showed weak to moderate staining for all the groups but IDC, where the diffusely infiltrated small and irregular epithelial cells showed strong staining at mRNA and protein level. Conversely, the stromal cells showed a weak pattern of staining at mRNA and protein levels (Figure 3A and 3J). Regarding the pre-neoplastic diseases analyzed, FLRG intensity in the epithelial cytoplasm and nucleus were not different from the control group. On the other hand, FLRG staining intensity was stronger in the epithelial cytoplasm of the IDC at mRNA (Figure 4C, P = 0.007) and protein (Figure 4F, P = 0.008) levels and in the epithelial nucleus for both mRNA (P = 0.016) and protein (P = 0.037). No statistically significant changes in the stromal intensity were observed in all the proliferative diseases analyzed (Figure 4G and 4H).

## Discussion

In the present paper we first demonstrated that human breast proliferative disorders such as FH, FIB and DCIS, express FST and FLRG and confirmed previous findings describing FST and FLRG expression in NB and IDC [17]. In the NB and in all the breast proliferative disorders analyzed, FST and FLRG were localized in the epithelial and in the stromal cells at mRNA and protein levels. These expressional patterns are similar to the one we [2,28] and others [4] previously described for activin in human normal and pathological breast tissues. Here we also found an increased expression of FST in stromal cells of FIB compared to NB and FLRG up regulation in IDC.

FIB are benign tumors of the breast typically composed of stromal and epithelial cells [29] and we previously reported that activin βA was down regulated in this breast disease [2]. Taken together, the findings that demonstrate down regulation of activin βA and up regulation of FST in FIB suggest that activin anti-proliferative effects may be weakened, thus favoring the cellular events that lead to the establishment of the FIB lesion. Additionally, in a mouse model, Krneta and co-workers (2006) [20] showed that although FST-expressing R30C tumors displayed increased angiogenesis, they were highly susceptible to undergo serum starvation-induced apoptosis, suggesting a role for FST in limiting tumor progression. Moreover, despite some controversy about the importance of hormonal effects in the development of FIB, some reports documented increased estradiol levels in the serum of women carrying FIB lesions [29]. In this connection, FST mRNA transcripts were decreased in the mammary gland of rats after ovariectomy, showing that FST expression is regulated by estrogen in the mammary gland [30].

Regarding FLRG, in the present study, we found that its expression was increased in IDC at protein level as previously reported [17] and that such increased expression was also present at mRNA level, implying that the tumor itself is the source of increased FLRG protein expression. Concerning activin's role in breast cancer, previously we reported an increased transcript profile of activin βA subunit and an augmented activin A concentration in homogenates of breast carcinoma [3]. On the other hand, in high grade breast cancer, an impairment of the activin signal transduction system has been linked to oncogenic progression, since a reduced expression of activin βB and its receptors, as much as alterations of smad signaling have been characterized [4]. All in all, together with the findings that FLRG contributes to tumor cell proliferation through antagonizing activin effects [17], we assume that the increased activin A expression reported in breast carcinoma is counteracted by an increased FLRG expression that prevents activin from binding its receptors and thus reducing smad signaling and activin anti-proliferative effects. Additionally, we also analyzed FLRG pattern of expression in pre-neoplastic diseases and in DCIS, a lesion where cancer cells do not infiltrate the adjacent stromal tissue and which has been pointed to be a precursor of IDC [31]. FLRG was only over-expressed in the IDC, suggesting that FLRG is strongly correlated with tumor progression and breast malignancy.

As for the cellular localization, the typical nuclear staining of FLRG already described in other cell types [32] was present in the normal and pathologic mammary glands evaluated here. We also observed FLRG staining in the cytoplasm, which recapitulates in vivo observations in human endometrium [21,25]. In the current study, FST staining was stronger in the cytoplasm, suggesting that the localization pattern of this protein in mammary gland epithelial cells is the same already described for other tissues [22,33]. Yet, we also noted nuclear staining of FST, which is rather atypical but has been described before in spermatogenic cells [34].

It is interesting to note that FST expression was increased in the stromal cells of FIB, while FLRG was up regulated in IDC, indicating that the two activin binding proteins may play diverse roles in tumor progression. Although their overall gene structure is quite similar, FST and FLRG seem to regulate activin pathway in different ways [35]. Indeed, in human liver tumor specimens, FST expression was increased in about 60% while FLRG transcript profile remained unchanged [18]. Conversely, in endometrial carcinoma, FST expression is unchanged while FLRG expression is down-regulated [21]. FLRG was also found to be down-regulated in ovarian endometriosis, while FST expression was found to be up-regulated [22]. Collectively, these finds demonstrate a number of differences between FST and FLRG, further suggesting that they may not be complete functional homologues.

Finally, a further mechanism to be hypothesized is that activin may have, similarly to TGFβ, a dual role on cancer progression. In early phase, TGF-β inhibits growth of cancer cells by cytostasis, differentiation and apoptosis. In later phase, instead, it works to promote cancer progression and metastasis by several mechanisms including evasion from immunological recognition, production of growth factors, differentiation into an invasive phenotype, metastatic dissemination as much as the establishment and dispersion of metastatic colonies [36,37]. Although whether activins work similar to TGF-β is not currently known, despite the anti-proliferative actions of activin, follistatin seems to act as an inhibitor of cancer metastasis [19].

## Conclusion

The current findings suggest a role for FST in breast benign disease, particularly in FIB, since its expression was increased in the stromal cells of this benign disease. Moreover, given that both FLRG mRNA and protein are up regulated in IDC, we conclude that the tumor is the source of the increased FLRG peptide in this invasive carcinoma. In addition, since FLRG expression is not altered in DCIS, but only in IDC, we conclude that FLRG may play an important role towards malignancy. As activin displays an anti-proliferative effect in human mammary cells, the present findings indicate that increased FST and FLRG expression in breast proliferative diseases might counteract the anti-proliferative effects of activin.

## Competing interests

The authors declare that they have no competing interests.

## Authors' contributions

EB, HLC, LM, PC, MM, and LEB carried out the experiments described in the study. VFA and GDC reviewed the pathological diagnosis of the breast samples while the present study was designed and analyzed by MM, GG, FP and FMR. All authors read and approved the final manuscript.

## Pre-publication history

The pre-publication history for this paper can be accessed here:

http://www.biomedcentral.com/1471-2407/9/320/prepub
